# Epidemiology and risk factors of patients with types of acute coronary syndrome presenting to a tertiary care hospital in Sri Lanka

**DOI:** 10.1186/s12872-019-1217-x

**Published:** 2019-10-21

**Authors:** Udaya Ralapanawa, Pallegoda Vithanage Ranjith Kumarasiri, Kushalee Poornima Jayawickreme, Prabashini Kumarihamy, Yapa Wijeratne, Madhushanka Ekanayake, Chandira Dissanayake

**Affiliations:** 1 Department of Medicine, University of Peradeniya, Peradeniya, Sri Lanka; 2 Department of Community Medicine, University of Peradeniya, Peradeniya, Sri Lanka; 3 Teaching Hospital Peradeniya, Peradeniya, Sri Lanka

**Keywords:** Acute coronary syndrome, Unstable angina, Non-ST elevation myocardial infarction, ST elevation myocardial infarction, Sri Lanka

## Abstract

**Background:**

Acute Coronary Syndrome (ACS) is one of the leading causes of death worldwide and studies have shown higher mortality rates and premature death in South Asian countries. The occurrence and effect of risk factors differ by type ofACS.Epidemiological studies in the Sri Lankan population are limited.

**Methods:**

This is a cross sectional descriptive study conducted at the Teaching Hospital Peradeniya, Sri Lanka among patients presenting with ACS. Data was collected by an interviewer administered structured questionnaire and epidemiological patterns and risk factors were analyzed.

**Results:**

The sample of 300 patients had a mean age of 61.3+/− 12.6 and male sex showed higher association with all three type of ACS compared to female with a *P* value of 0.001. This study showed higher mean age of 62.2 ± 11.4 years amongst unstable angina (UA) patients and 61.9 ± 14.5 years amongst non ST elevation myocardial infarction (NSTEMI) patients compared to 59.2 ± 11.2 years for ST elevation myocardial infarction (STEMI) patients with no significant statistical difference (*P* = 0.246). Approximately 55.8% STEMI patients, 39.8% UA and 35.5% NSTEMI patients were smokers indicating a significant association between smoking and STEMI (*P* = 0.017). Nearly 54.5% STEMI, 35.4% UA and 32.7% NSTEMI patients consumed alcohol and there was a very strong association between alcohol consumption and STEMI (*P* = 0.006). Almost 51.8% NSTEMI patients, 47.8% UA patients and 29.9% STEMI patients had hypertension(HT) (*P* = 0.008) indicating significant association of HT with UA and NSTEMI. About 33.6% UA patients and 30.0% NSTEMI patients had DM whilst only 22.1% of STEMI patients had DM of no significance (*p* = 0.225). Around 15.0% patients with UA, 25.5% with NSTEMI and 11.7% with STEMI had dyslipidemia (*P* = 0.032). There was a very strong association between a past history of ACS or stable angina with NSTEMI and UA (*P* = 0.001).

**Conclusion:**

Smoking and alcohol abuse are significantly associated with STEMI.Patients with NSTEMI or Unstable Angina had higher rates of hypertension and were more likely to have a history of ACS or stable angina than STEMI patients. Patients with NSTEMI were more likely than patients with STEMI or UA to have dyslipidemia.

## Background

Ischaemic heart disease (IHD)is the most common form of heart disease and the single most important cause of premature death worldwidedespite major breakthroughs in management [1]. According to the World Health Organization, IHD was responsible for 8.1 million deaths worldwide in 2013 (95% uncertainty interval, 7.3–8.8 million) and there was an increase of 42% in the number of IHD deaths since 1990.

According to the Annual health bulletinof Sri Lanka in2013 increasing trend was seen in hospitalizations per 100,000 population due to ischaemic heart diseases; 494.9 in 2012 and 506.1 in 2013. Ischemic heart disease isranked as the leading cause of hospital deaths in Sri Lanka since 1995 having great clinical and financial impact. It accounted for 29.1 deaths per 100,000 population in 2013, including 14.7% hospital deaths with a case fatality rate of 5.76 [1].

Acute coronary syndrome (ACS) includes Unstable Angina (UA) and evolving Myocardial Infarction (MI) which is usually divided into ST-segment elevation Myocardial Infarction (STEMI) or new onset Left Bundle Branch Block (LBBB), and ACS without ST-segment elevation (NSTEMI) [2].

Several studies have been published on epidemiology, risk factors andthe outcome of ACS in Western countries. Studies have shown that South Asians experience higher mortality rates and premature deaths accounting to IHD (deaths occurring at least 10–15 years before expected) than is experienced by people in Western countries [3, 4]. However, there are only limited studies which relate to IHD in the Sri Lankan population.

ACS often reflects a degree of damage to the coronary arteries by atherosclerosis; plaque rupture, thrombosis, and inflammation. Advanced age, male gender and afamily history of ischemic heart disease have been identified as non-modifiable risk factors.Smoking, hypertension (HT), diabetes mellitus (DM), dyslipidemia, obesity and a sedentary lifestyle were identified as modifiable risk factors [2]. However, the data is limited on the association of family history of non-communicable diseases and ACS. The risk factors contributing to the development of IHD were summarized by the Framingham Heart Study providing crucial information regarding intervention for primary and secondary prevention of IHD [5].

According to previousstudies,the management of IHD in developing countries is generally suboptimal compared to developed countries. This is true for Sri Lanka and regionally [6–8]. Even though clinical trials provide evidence of current management practices(medication and intervention) for diseases, only observational studies provide details of epidemiology, risk factors, shortcomings in management and differences in outcomes among different regions of the world and within the country itself [9, 10]. However, since Sri Lanka does not currently have a cardiac registry the outcomes of ACS following the index admission are not known.

The aim of the current study is to determine the epidemiology and risk factors of patients with Acute Coronary Syndrome presenting to a tertiary care hospital in Sri Lanka.

## Methods

### Study type

A cross sectional descriptive study was performed among patients diagnosed with ACS presenting to the medical ward, Teaching Hospital Peradeniya.

### Sample population and inclusion, exclusion criteria

This study sample consists of a total of 300 patients who presented to the medical ward of the Teaching Hospital Peradeniya with ACS over a period of one year (July 2014 onwards). This includes patients who were between 20 and 85 years of agecategorized into the three types of ACS; STEMI, NSTEMI and UA. They were diagnosed and classified according to the American College of Cardiology/ American Heart Association (ACC/AHA) definitions and were treated as per ACC/AHA recommendations [11, 12]. UA was diagnosed with the presence of at least one of the following criteria: angina usually lasting for ≥20 min, onset within one month or angina occurring within a crescendo pattern. Patients with UA also had at least one of the following electrocardiogram (ECG) findings: ST segment depression ≥0.5 mm or T inversion ≥0.3mv in any two leads. NSTEMI was diagnosed with the presence of elevated Troponin I levels as an indicator of myocardial necrosis in addition to other features of UA. STEMI was diagnosed with the presence of clinical symptoms of MI lasting ≥30 min with ECG changes of either ST elevation of at least 0.1mv in two contiguous precordial leads or two limb leads, or the presence of a new LBBB. Those with either myocarditis, pericarditis or endocarditis, patients with pre-existing structural heart disease, connective tissue disorders and pregnant patients were excluded from this study.

Risk factors of each of these patients were identified. Dyslipidemia was identified with patients either taking lipid lowering drugs or biochemically indicated by total cholesterol > 240 mg/dl, triglycerides >150mh/dl, low density lipoproteins (LDL) > 130 mg/dl and high-density lipoproteins (HDL) < 50 mg/dl, < 40 mg/dl for females and males respectively. Diabetes mellitus was defined as either with a history of the subject having the diagnosis or currently being on treatment or fulfilling diagnostic criteria for diabetes mellitus according to the current American diabetes Association (ADA) guidelines [presence of clinical symptoms and plasma glucose concentration ≥ 200 mg/dl (11.1 mmol/l) or fasting blood sugar ≥126 mg/dl (7.0 mmol/l)]. Hypertension was diagnosed with systolic blood pressure ≥ 140 mmHg and/ or diastolic blood pressure ≥ 90 mmHg and/or being on antihypertensive treatment. Family history of ACS was defined as a first degree male relative withIHD which developed below 55 years of age or a first degree female relative with IHD which developed below 65 years of age. In addition, the family history of other risk factors like ischaemic stroke or transient ischaemic attack, hypertension, dyslipidemia and DM were assessed. Other modifiable risk factors like smoking, alcohol consumption and social stressors were also assessed. The clinical presentation of the patients was assessed on admission and they were followed up daily during their hospital stay while assessing their management and outcomes.

### Study setting

Teaching Hospital Peradeniya is a tertiary care hospital situated in the Central Province of Sri Lanka. It consists of 800 bedsincluding Professorial Teaching units in Medicine, Surgery, Pediatrics and Obstetrics and Gynaecology. Patients with ACS will either present directly to the medical ward or to the Preliminary Care Unit where initial emergency managementis doneincluding administration of Streptokinase. Patients will subsequently be transferred and treated in the High Dependency Care Unit during the initial days and once stabletransferred to the medical ward. An intensive care unit facility is available within the hospital in case of emergency. Patients who require further specialized cardiac management including percutaneous coronary intervention are transferred to the closest specialized coronary care unit at the Teaching Hospital Kandy which can be reached within ten minutes by ambulance.

### Data collection

Data collection commenced following approval from the Institutional Ethical Review Committee (IERC) of the Faculty of Medicine, University of Peradeniya. A pilot study was conducted on 30 individuals prior to data collection to finalize the questionnaire. Participants selected for the study were interviewed after informed written consent was obtained. Data was collected by an interviewer administered structured questionnaire (Additional file 1) byqualified doctors who were specially trained for this research.Changes in ECGs and Troponin I results were collected from bed head tickets. Confidentiality was maintained throughout the study including the storage and analysis of data.

### Data analysis

The data wascoded and entered into an excel data sheet and was analyzed using SPSS, version 20.0 (IBM, Armonk, NY, United States of America). Aunivariate analysis was conducted initially and for selected variables a bivariate analysis was conductedsubsequently. The *X*^*2*^ test for nominal scale data was used to identify statistical significance. One way analysis of variance (ANOVA) was used to compare differences in age between three ACS groups. As the *P* value was non-significant, no post hoc tests (Bonferroni test) were used. Probability of less than 0.05 was used to ascertain statistical significance.

## Results

The total sample consisted of 300 patients presenting with ACS, with a mean age of 61.3+/− 12.6. This included 199(66.3%) males (mean age 60.1+/− 12.2) and 101(33.7%) females with a mean age of 63.8+/− 12.9 (*p* = 0.014). The male: female ratio was approximately 2:1. The majority of the patients were Sinhalese in ethnicity (84.0%) (Table 1).

**Table 1:** Patient baseline characteristics according to the type of ACS

variable	UA	NSTEMI	STEMI	*P* value
Number of Patients	113(37.7%)	110(36.7%)	77(25.7%)	
Age(years)mean	62.2 ± 11.4	61.9 ± 14.5	59.2 ± 11.2	0.246
RaceSinhalese	98(86.7%)	91(82.7%)	63(81.8%)	
Tamil	5(4.4%)	5(4.5%)	4(5.2%)	
Muslim	10(8.8%)	14(12.7%)	10(12.9)	
Type of chest pain				
Left	45(39.8%)	36(32.7%)	22(28.6%)	0.021
Right	12(10.6%)	9(8.2%)	7(9.1%)	0.513
Central	48(42.5%)	60(54.5%)	45(58.4%)	0.282
Diffuse	5(4.4%)	4(3.6%)	2(2.6%)	
Epigastric	3(2.7%)	1(0.9%)	1(1.3%)	
Faintishness	35(30.9%)	31(28.2%)	25(32.5%)	0.807
Past medical history				
Diabetes Mellitus	38(33.6%)	33(30.0%)	17(22.1%)	0.225
Hypertension	54(47.8%)	57(51.8%)	23(29.9%)	0.008
Dyslipidemia	17(15.0%)	28(25.5%)	9(11.7%)	0.032
ACS/stable angina	52(46.0%)	53(48.2%)	18(23.4%)	0.001
Family history				
ACS	37(32.7%)	41(37.3%)	21(27.3%)	0.358
Stroke	14(12.4%)	12(10.9%)	11(14.3%)	0.787
Hypertension	36(31.9%)	39(35.5%)	18(23.4%)	0.207
Diabetes Mellitus	33(29.2%)	39(35.5%)	18(23.4%)	0.202
Dyslipidemia	21(18.6%)	27(24.5%)	17(22.1%)	0.555

**Fig. 1:**
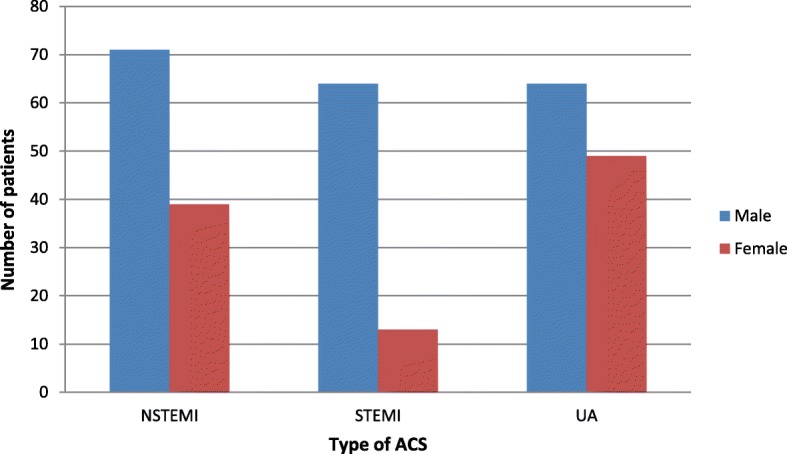
Gender distribution according to the type of Acute Coronary Syndrome

**Fig. 2:**
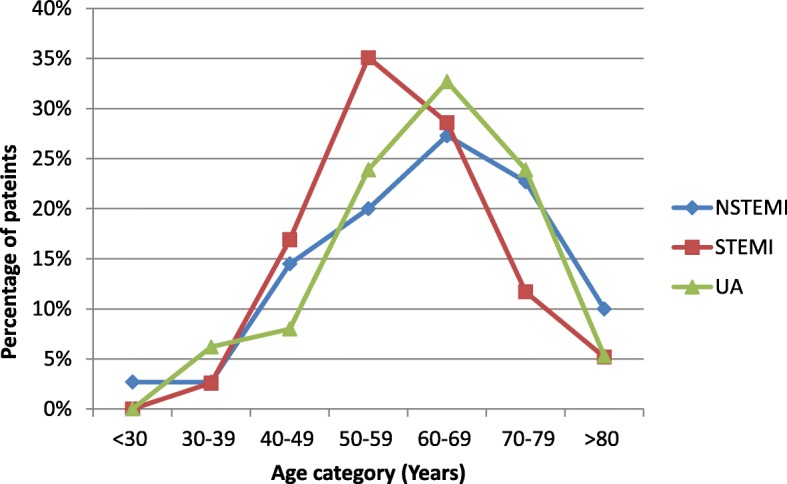
Age distribution of the sample according to the type of Acute Coronary Syndrome

Out of 300 diagnosed acute coronary syndrome patients 113(37.7%) had unstable angina, 110(36.7%) NSTEMI and 77(25.7%) had STEMI. Of the total NSTEMI patients a majority of 64.5%(71) were male, whereas a more significant majority of 83.1%(64) of the STEMI patients were male. A further 56.6% (64) of unstable angina patients were male. Thusthe male sex showed a higher association with all three type of ACS with a *P* value of 0.001 (Figure 1). Almost 2.7% of NSTEMI patients were below 30 years and none of the STEMI or unstable angina patients of this population were below 30 years. The highest percentage of NSTEMI and unstable angina patientsbelong to the 60–69 year age group accounting for 27.3 and 32.7% respectively, whilst the highest percentage of STEMI patients (35.1%) were between 50 and 59 years of age (*p* = 0.144) (Fig. 2). Our study showed a higher mean age of 62.2 ± 11.4 yearsin UA patients and 61.9 ± 14.5 years in NSTEMI patients compared to 59.2 ± 11.2 years for STEMI patients, with no significant statisticaldifference (*P* = 0.246) (Table 1).

The presentation of chest pain was either left, right, central, diffused or epigastric. A majority of 54.5% NSTEMI patients and 58.4% STEMI patients presented with typical retrosternal central chest pain. This figure for unstable angina was 42.5%. About 2.7% of UA patients, 1.3% of STEMI patients and 0.9% of NSTEMI patients presented with epigastric pain. Approximately 28.2% of NSTEMI patients, 32.5% STEMI patients and 30.9% of UAhad faintishness on admission (*p* = 0.807) (Table 1).

**Fig. 3:**
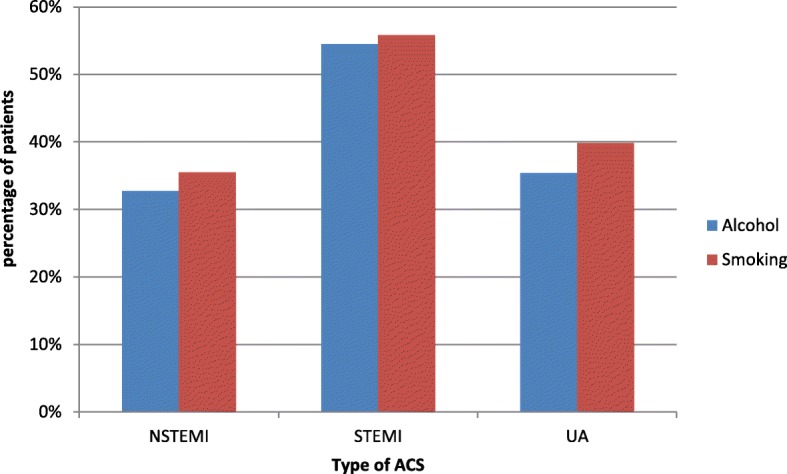
Association of alcohol and smoking with the type of Acute Coronary Syndrome

Out of 300 patients 127(42.3%) were regular smokers and the mean number of cigarettes per day was 3.85+/− 6.5 and the mean duration of smoking was 9.6+/− 13.8 years. 55.8% STEMI patients were smokers whereas only 39.8% UA and 35.5% NSTEMI patients were smokers indicating a significant association between smoking being a risk factor for STEMI compared to NSTEMI and UA (*p* = 0.017) (Fig. 3). Nearly 39.3% (118) patients consumed alcohol and a majority of 54.5% STEMI patients and, only 35.4% UA and 32.7% NSTEMI patients consumed alcohol indicating a very strong association between alcohol consumption and STEMI (*p* = 0.006)(Fig. 3). Nearly 32.4% UA, 22.6% NSTEMI patients and 13.7% STEMI patients had social stressors and there was a significant statisticalassociation of social stressors (*p* = 0.013) with UA compared to the other two types.

Among 300 patients 88 (29.3%) had DM, 134 (44.7%) had HT, 123 (41.0%) had a prior history of acute coronary syndrome or stable angina and 54 (18.0%) had dyslipidemia. 51.8% NSTEMI patients, 47.8% UA patients and 29.9% STEMI patients had HT (*P* = 0.008) indicating a significant association between HT and UA and a more significant association with NSTEMI. Around 33.6% UA patients and 30.0% NSTEMI patients had DM whilst only 22.1% of STEMI patients had DM (*p* = 0.225) without reaching any significant association between DM and type of ACS. Nearly 15.0% patients with UA, 25.5% with NSTEMI and 11.7% with STEMI had dyslipidemia and showed a slightly significant association between dyslipidemia and NSTEMI (*P* = 0.032). Almost 48.2% patients with NSTEMI, 46.0% patients with UA and 23.4% patients with STEMI had a past history of ACS or stable angina. This indicates a very significant association between a past history of ACS or stable angina with NSTEMI and UA (*P* = 0.001) compared to STEMI (Table 1).

Out of 300 patients 99 (33.0%) had a family history of ACS, 90 (30.0%) had a family history of diabetes mellitus, 93 (31.0%) had a family history of hypertension, 37(12.3%) had a family history of strokes and 65 (21.7%) had a family history of dyslipidemia. About 37.3% of NSTEMI, 32.7% UA and 27.3% of STEMI patient had a family history of ACS whereas 14.3% STEMI, 12.4% UA and 10.9% of NSTEMI patients had a family history of stroke. The presence of family history of ACS (*P* = 0.358) or stroke (*P* = 0.787) was not significantly associated with the type of ACS. Approximately 35.5% NSTEMI patients, 23.4% STEMI patients and 29.2% UA patients had a family history of DM whilst 35.5% NSTEMI patients, 23.4% STEMI patients and 31.9% UA patients had a family history of HT.Therewas no significant statisticalassociation between family history of DM or HT with the type of ACS (*P* = 0.202 and 0.207 respectively). 24.5% NSTEMI patients, 22.1% STEMI patients and 18.6% UA patients had a family history of dyslipidemia (*P* = 0.555) indicating no significant association with the type of ACS(Table 1).

On admission the majority of 299 patients (99.7%) used chewable or dissolved aspirin as an anti-platelet agent. Out of 300, 278(92.7%) patients recovered, 20(6.7%) were transferred to a coronary care unit for further management and 2(0.7%) died after initial management. Mean hospital stay was 4.2+/− 1.3 days and all patients were given primary health education on discharge.

## Discussion

Epidemiological studiesin South Asian countries have led to important conclusions regarding the prevalence, the type of presentation and the treatment of ACS in the region [3, 4, 8, 13]. These studies also showed geographic differences and heterogeneity in the care and mortality of patients with ACS throughout Asia. However studies regarding epidemiology and risk factors of types of ACS in the Sri Lankan population are limited.

The ACCESS group of investigators reported 46% ACS in developing countries to be STEMI and 54% to be NSTEMI/UA [14]. *Rajapakse* et al.in 2010 reported 33.6% ACS in Sri Lanka to be STEMI [15], while in 2012 *Medagama* et al reported 32.8% ACS to be STEMI [13], while our study in 2014 showed 25.7% to be STEMI, 36.7% NSTEMI and 37.7% UA.

South Asians show a higher rate of MI at a younger age (mean age 53 years) compared to those in other countries (mean age 58.8 years). This is very likely due to South Asians having more risk factors at ages < 60 years when stratified by age, including higher apolipoprotein B_100_ /apolipoprotein A-I ratio and higher waist to hip ratio [16]. *Sharma* et al alsoshowed that ACS occurred a decade earlier in South Asians compared to the Western population [17]. However, our study showed a mean age of presentation of ACS in Sri Lanka to be around 61 while it was 63.7 years according to *Medagama* et al in 2012 [13]. Therefore, Sri Lankans develop ACS later in their life compared to other South Asians. Larger scale studies within the region are needed to explain this difference.

Our study showed that the mean age for females (64 years) was significantly higher (*p* = 0.014) compared to males (60 years). Studies have shown that the onset of cardiovascular disease in women occurs seven to ten years after that for males [18]. Exposure to endogenous oestrogens during the premenopausal period are assumed to delay the manifestation of atherosclerotic disease in females, likely due to oestrogens having a regulatory effect on lipids, inflammatory markers, coagulant system and promoting a direct vasodilatory effect through α and β receptors in vessel walls [19, 20].

Our study showed that the highest proportion of 27.3% NTEMI patients and 32.7% UA patients were between 60 and 69 years of age and the highest percentage of 35.1% STEMI patients were aged between 50 and 59 years with no significant statistical difference in age distribution with the type of ACS.*Medagama* et al also showed no significant difference in age distribution of patients with all groups of ACS, with the majority being between 51 and 70 years of age [13]. *Sharma* et al showed a higher mean age of 60.07 ± 10.47 years amongst NSTEMI patients compared to 57.76 ± 11.44 years for STEMI patients with no significant statisticaldifference (*p* = 0.103) [17]. Similarly, our study also showed a slightly higher mean age for UA (62.2 years) and NSTEMI (61.9 years) compared to STEMI (59.2 years) but without a statistical significance (*P* = 0.246).

In our studynearly a two third (66.3%) of patients with ACS were maleand themale gender was strongly associated with all three type of ACS compared to the female gender. A study done in Nepal also showed astrong association of ACS with male(75.7%) [21]. This male preponderance is seen in theINTERHEART study (overall male 76%) and its South Asian cohort(85%) [22]. In our study a higher percentage of 48.5% females had UA compared to NSTEMI (38.6%) and STEMI (12.9%) indicating a higher risk for UA than NSTEMI and STEMI in females (*P* = 0.001).However males did not show a preponderance for any type of ACS.

The INTERHEART study describes nine risk factors which account for > 95% of acute MI. Eight risk factors including dyslipidemia, smoking, hypertension, DM, abdominal obesity, psychosocial factors, lack of consumption of fruits, vegetables and of regular physical activity were significantly associated with MI (*p* < 0.0001), whereas alcohol consumption had a weaker association (*p* = 0.03). Multivariate analysis showed the two strongest risk factors to be current smoking and increased apolipoprotein B_100_ /apolipoprotein A-I ratio, followed by a history of DM, HT and psychosocial factors. Rare novel risk factors are high inflammatory markers and homocysteine levels [22].

Hypertension was the most common risk factor with 134 (44.7%) patients being hypertensive. According to the INTERHEART study the prevalence of hypertension in the South Asian cohort was 31.1% which is much lower than our study. However, a study done in India [8] reported 48.8% HT and another study showed 40.2% [17] of Indians with ACS to be hypertensive similar to our study. Howeverthe ACCESS group of investigators reported a higher prevalence of HT (STEMI 78%, NSTE-ACS 87%) in ACS patients in other developing countries compared to Sri Lanka and India [14]. Further, hypertension was significantly more common in NSTEMI and UA patients than STEMI patients in our study.*Medagama* et al also showed HT to be more common in NSTEMI/UA patients [13]. Similarly, *Sharma* et al showed HT to be significantly associated with NSTEMI [17]. Sympathetic hyperactivity in HT contributes to higher risk of sudden death, coronary spasm and thrombosis. Chronic HT causes left ventricular hypertrophy leading to increased oxygen demand which results in the formation of collaterals. These collaterals are more effective in the subepicardial layer than the subendocardial layer resulting in partial thickness infarction. Reduction in diastolic blood flow leads to ischemia without total vessel occlusion [23]. These could explain HT more likely to be a risk for NTEMI and UA than for STEMI.

Smoking is an established risk factor for IHD and nearly 30% of all deaths from ACS were found to be attributed to smoking [24]. Alcohol abuse is a risk factor for occurrence of early ACS and patients with a combination of alcohol abuse and smoking had an even higher risk of developing very early ACS than those with the two individual risk factors alone [25]. In our study out of 300 patients, 127(42.3%) were regular smokers being the second most common risk factor and 118 patients (39.3%) consumed alcohol. Our study further showed smoking (*p* = 0.017) and alcohol consumption (*p* = 0.006) to be more significantly associated STEMI than for NSTEMI and UA. Similarly, *Medagama* et al and *Sharma* et al alsoshowed smoking and alcohol abuse to be more common in STEMI patients [13, 17]. Current smoking is an independent predictor of atherosclerotic plaque burden. Smoking causes plaque instability with a thin plaque cap leading to increased plaque rupture and superadded thrombosis due to the prothrombotic effect of smoking, which can explain the likely presentation of STEMI than of NSTEMI in smokers [26, 27].

Nearly 41% of patients had a prior history of ACS or stable angina in contrast to a study in India reporting only 20.7% having a prior history of ACS [8]. This could probably be due to patients with a past history of ACS events being on proper medical follow up and having modified or controlled other risk factors leading to ACS though a past history of ACS may also be a significant risk factor. Past history of ACS or stable angina were significantly more common inNSTEMI and UA patients than STEMI patients. This is similar to results reported by *Medagama*et al [13]. A study on recurrent MI showed a majority of 76% of recurrent MI to be of the same type of MI. Recurrent STEMI occurred in 44%, recurrent NSTEMI in 32%, and STEMI and NSTEMI in 24% [28].

Diabetes is another important risk factor which was found in 29.3% of the study population compared to a study in India reporting 35.5% having DM [8]. Interestingly, DM did not show a significant association with the type of ACS in our study in contrast to *Sharma* et al (showed DM to be significantly associated with NSTEMI) [17]. Hyperglycemia inhibits production of vasodilators like nitric oxide, and increases vasoconstrictors like endothelin-1. Impaired insulin mediated skeletal muscle uptake of free fatty acids increases synthesis of Very Low-Density Lipoprotein (VLDL) and cholesteryl esters in the liver. Diabetic patients with ACS commonly have a combination of elevated triglycerides and low HDL than elevated total and LDL cholesterol levels, and this frequently leads to multivessel coronary artery atherosclerosis. Procoagulability, impaired fibrinolysis and formation of advanced glycation end products are other mechanisms of DM being a risk factor for ACS [29]. *Donahoe et al* showed diabetics with STEMI to have a higher one year mortality, and the one year mortality for non-diabetics with STEMI to be similar to that of diabetics with NSTEMI/UA [30].

Among the strongest predictors of in-hospital deaths in STEMI patients were found to be untreated dyslipidemia, as total cholesterol and LDL levels undergo significant alterations post-MI, with a change of 7 and 10% in STEMI and 5 and 6% in UA respectively [31]. Our study reported 18% ACS patients to have dyslipidemia with significant association with NSTEMI compared to STEMI/UA. Only 7% of ACS patient had dyslipidemia in a previous Sri Lankan study [13] whereas an Indian study showed 37.96% to have dyslipidemia.

The presence of family history of ACS, DM, HT, Dyslipidemia or stroke did not show statistically significant association with the type of ACS.

Many studies have been done on risk factors for ACS in the West according to which most management and prevention protocols have been carried out. This study as in some other studies in the South Asian population shows that the epidemiology and risk factors for ACS differs from that of the Western world. Thus lifestyle modifications for prevention and management of ACS should be altered and implemented according to Sri Lanka. The effect and occurrence of certain risk factors differ according to type of ACS. STEMI is known to have a higher mortality rate than NSTEMI/UA [14]. Fortunately according to our study, risk factors most likely to lead to STEMI are modifiable risk factors like smoking and alcohol abuse which can be easily limited or eliminated by proper patient education and lifestyle modifications. Risk factors like HT and dyslipidemia which were found to be more associated with NSTEMI can also be optimized and may probably benefit with different cut-off target levels in patients with STEMI,UA and NSTEMI. Since risk factors like a past history of ACS and a family history of ACS cannot be changedthese patients may also benefit with lower cut-off target levels of other controllable risk factors. Further studies can be done to further assess the pathophysiology of each of the risk factors to cause STEMI, NSTEMI or UA by which newer drugs targeting the pathway can be used to treat patients with each of the risk factors and ACS than treating the risk factor alone.

## Conclusion

The occurrence and effect of risk factors differ by type of ACS. Modifiable risk factors like smoking and alcohol abuse are significantly associated with STEMI. Patients with HT and a past history of ACS or stable angina are more likely to develop NSTEMI/UAthan STEMI whereas patients with dyslipidemia are more likely to develop NSTEMI than STEMI/ UA. Patients with DM do not have a preponderance for any type of ACS. More studies are required to understand epidemiology, presentation and risk factors of ACS particularly in regional levels as they can differ from one region to the other. This understanding would help to implement preventive measures including lifestyle modification and drug treatment optimizing risk factors.

## Supplementary information

**Additional file 1:** The English Version of the Questionnaire used for data collection.

## Data Availability

All the data are stored in password protected computer and hard copies also under the custody of the corresponding author. Data can be seen by request to the corresponding author.

## Abbreviations

ACC: American college of cardiology
ACS: Acute coronary syndrome
AHA: American Heart Association
CVD: Cardiovascular disease
DM: Diabetes mellitus
ECG: Electrocardiogram
HDL: High density lipoprotein
HT: Hypertension
LBBB: Left bundle branch block
LDL: Low density lipoprotein
MI: Myocardial infarction
NSTEMI: Non-ST elevation myocardial infarction
STEMI: ST elevation myocardial infarction
UA: Unstable angina
VLDL: Very low density lipoprotein
