# Supplementary figures and images for: Epidemiology and risk factors of patients with types of acute coronary syndrome presenting to a tertiary care hospital in Sri Lanka
Source: BMC Cardiovasc Disord. 2019 Oct 21;19:229. doi: 10.1186/s12872-019-1217-x (PMC6805431; doi:10.1186/s12872-019-1217-x)

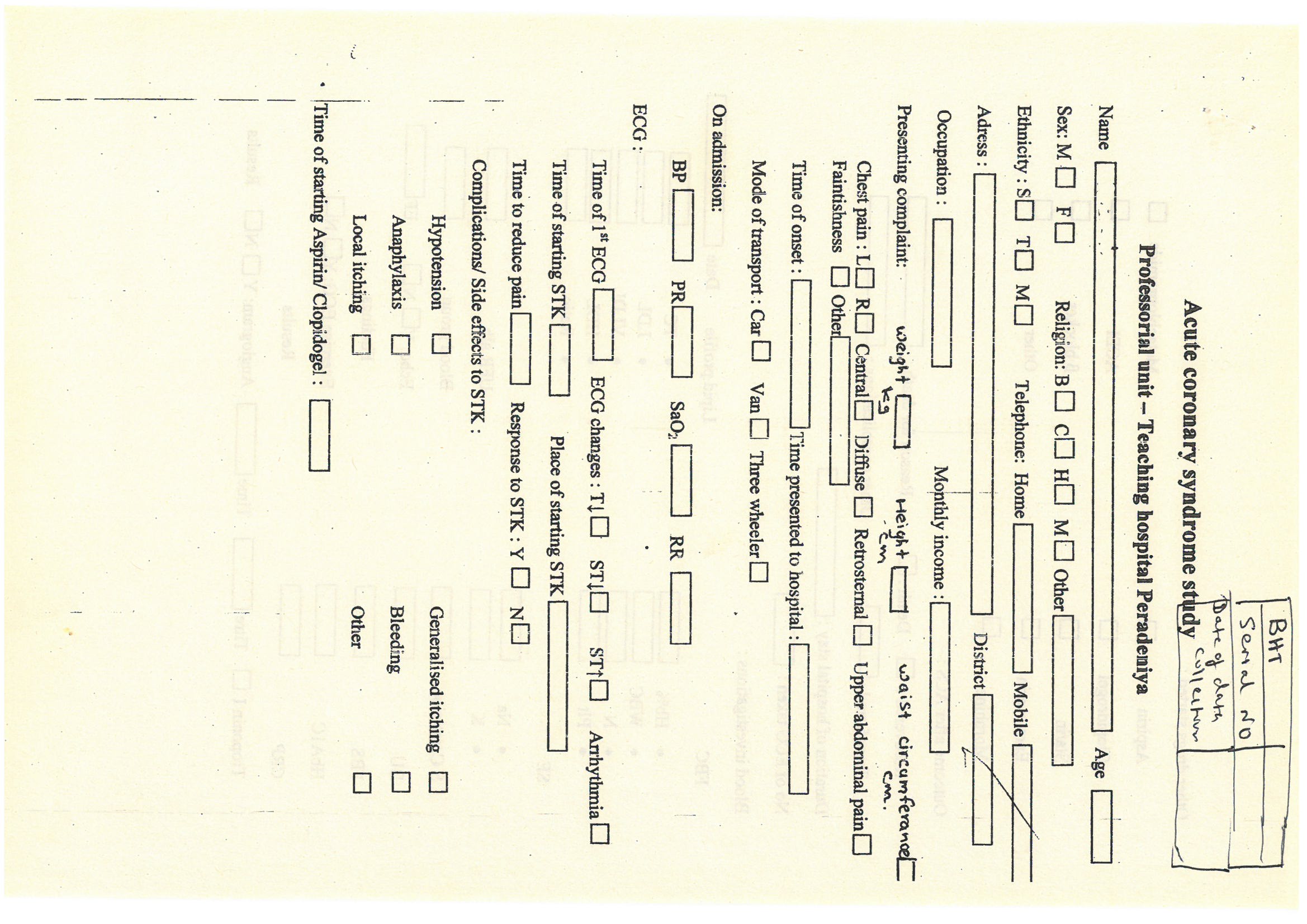

Supplement: Supplementary file 1 — Additional file 1: The English Version of the Questionnaire used for data collection. [file 12872_2019_1217_MOESM1_ESM.zip › Q page 1R3.jpg]

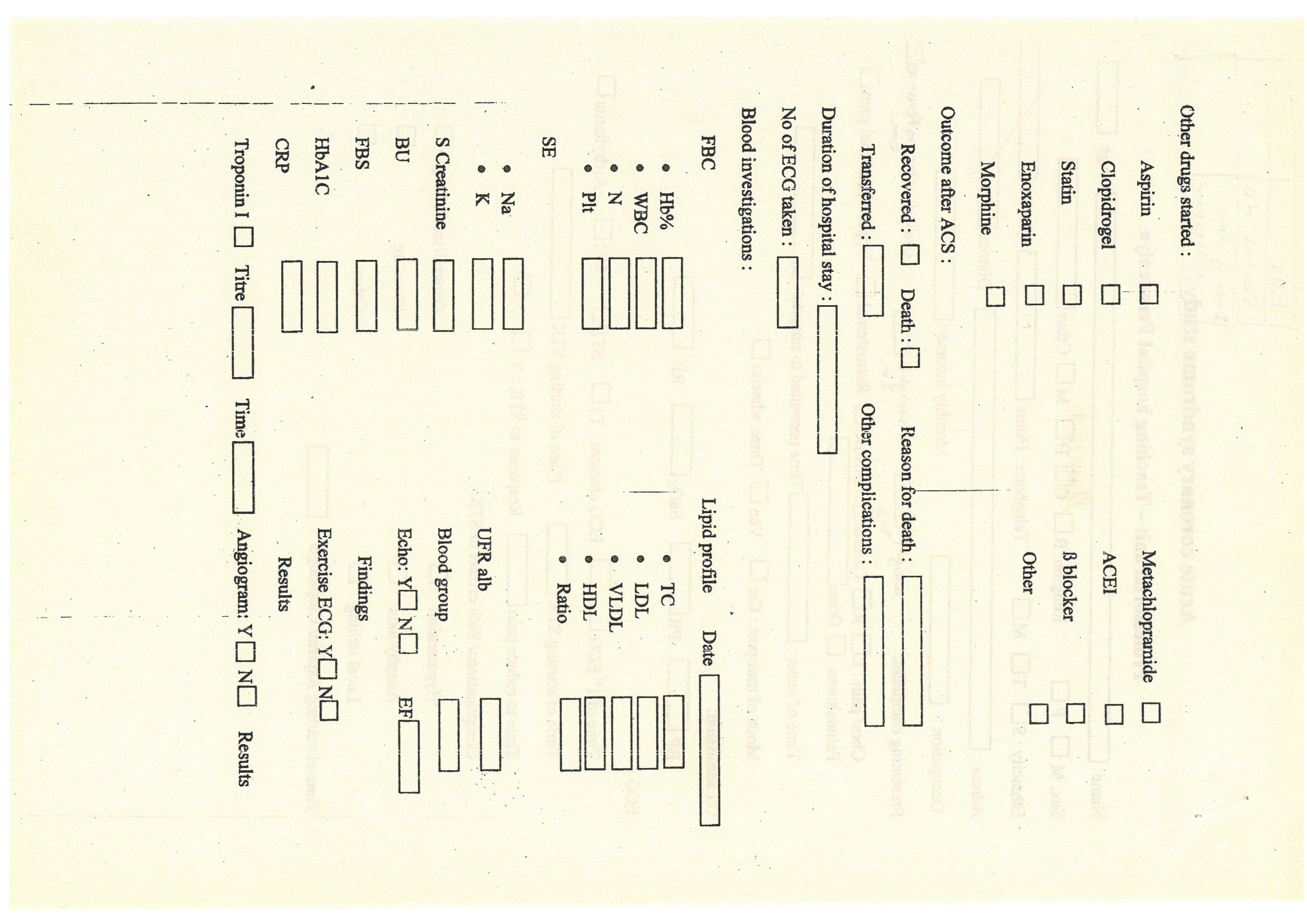

Supplement: Supplementary file 1 — Additional file 1: The English Version of the Questionnaire used for data collection. [file 12872_2019_1217_MOESM1_ESM.zip › Q Page 2R3.jpg]

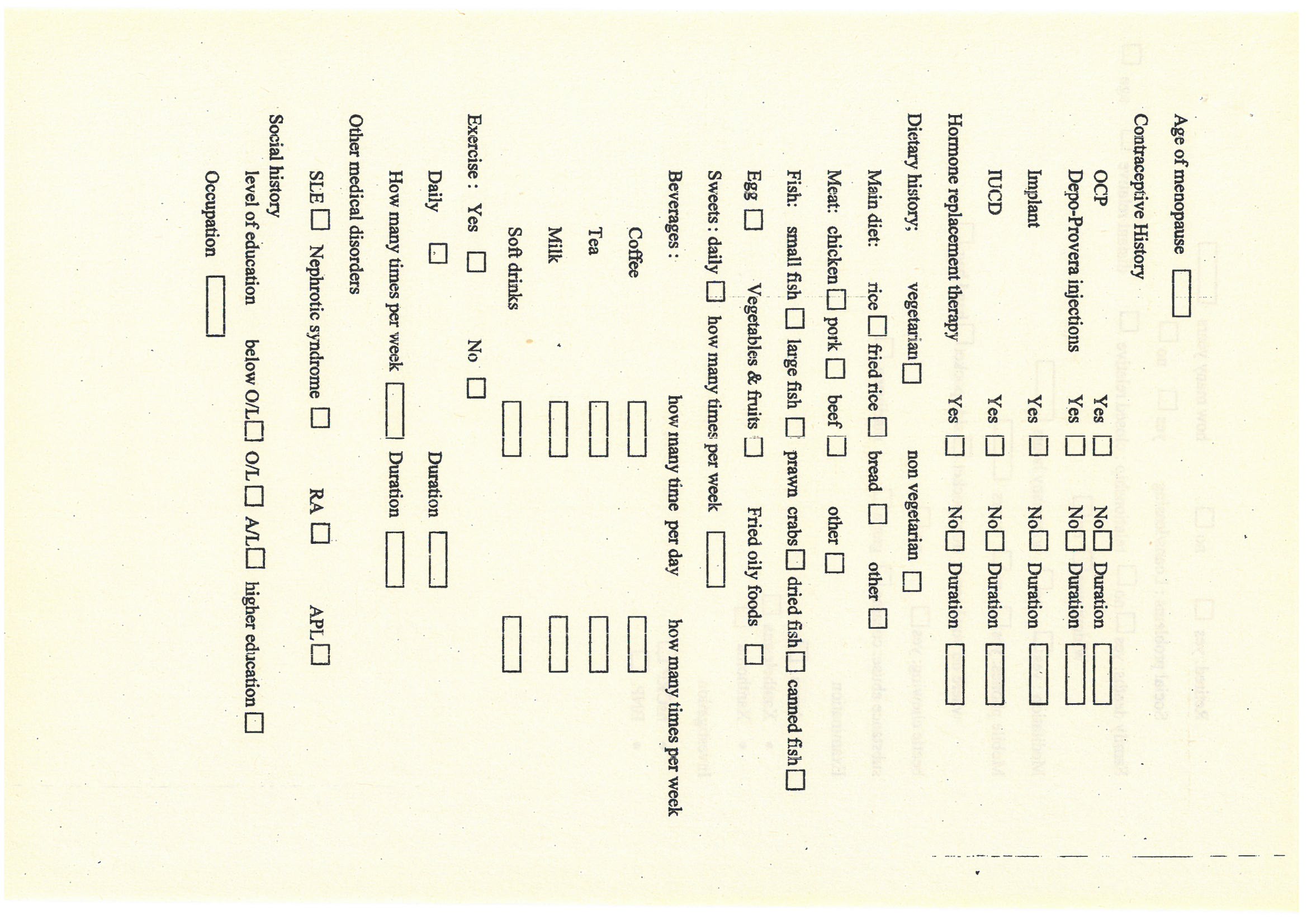

Supplement: Supplementary file 1 — Additional file 1: The English Version of the Questionnaire used for data collection. [file 12872_2019_1217_MOESM1_ESM.zip › Q Page 3R3.jpg]

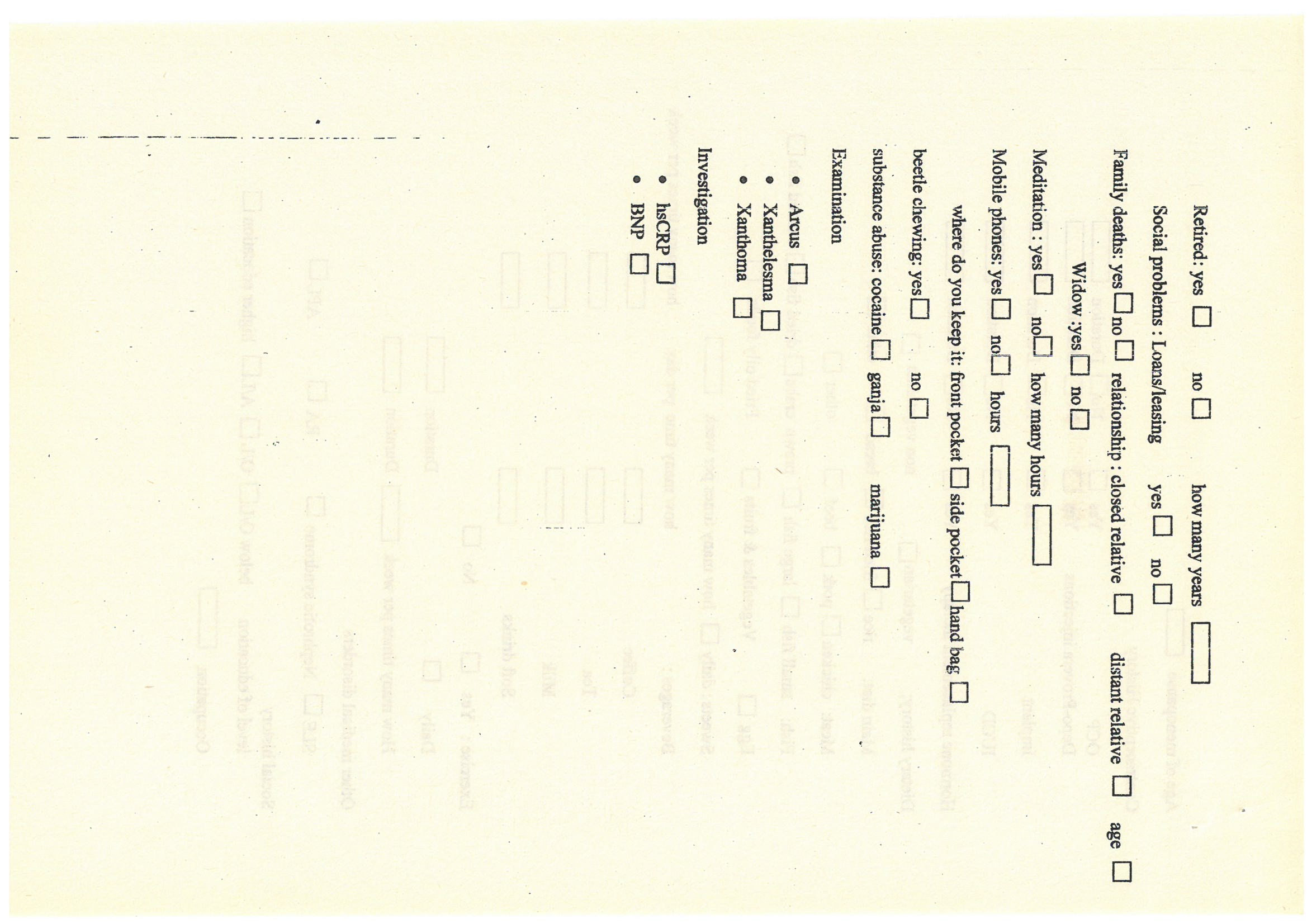

Supplement: Supplementary file 1 — Additional file 1: The English Version of the Questionnaire used for data collection. [file 12872_2019_1217_MOESM1_ESM.zip › Q Page 4R3.jpg]

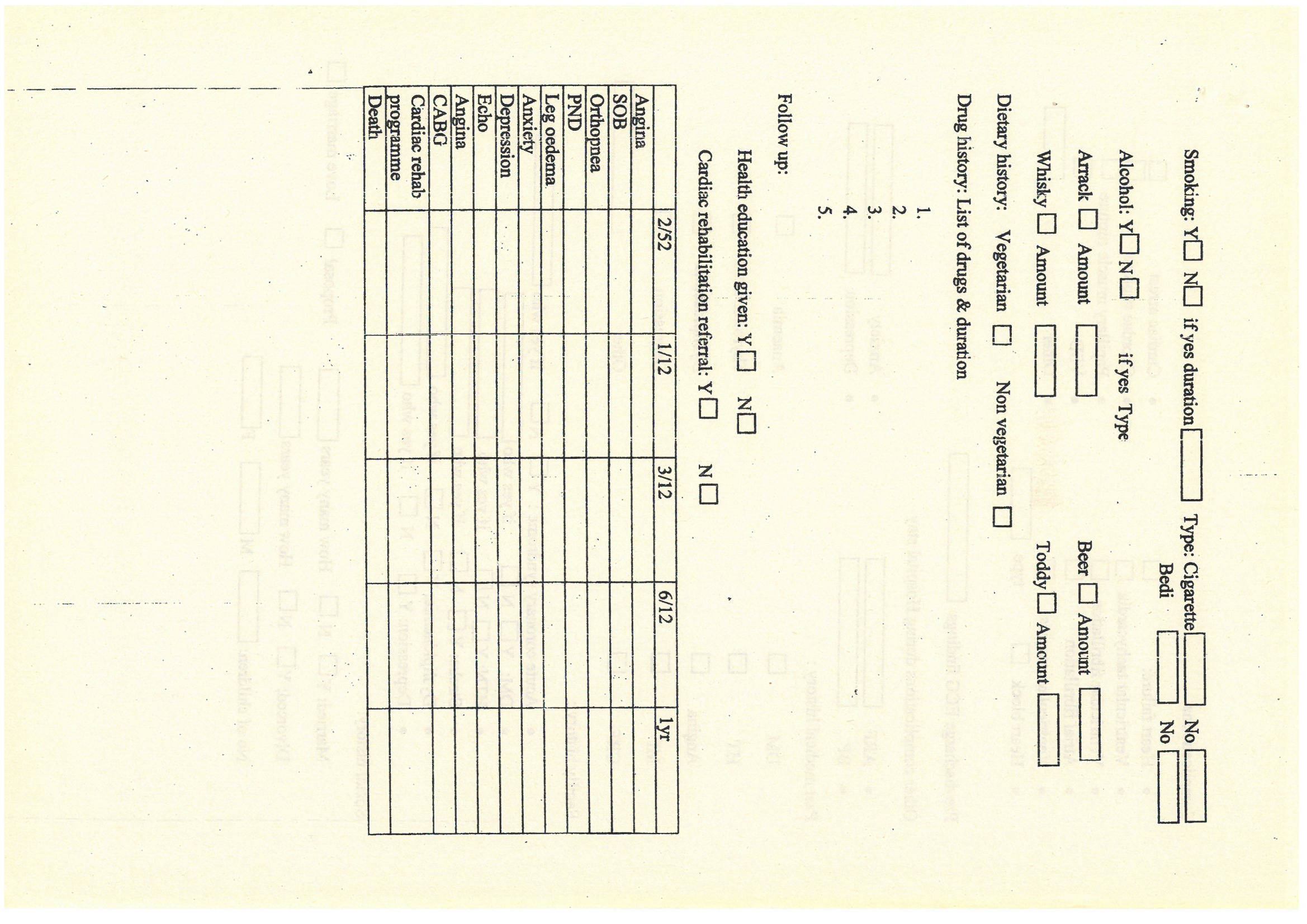

Supplement: Supplementary file 1 — Additional file 1: The English Version of the Questionnaire used for data collection. [file 12872_2019_1217_MOESM1_ESM.zip › Q Page 5R3.jpg]

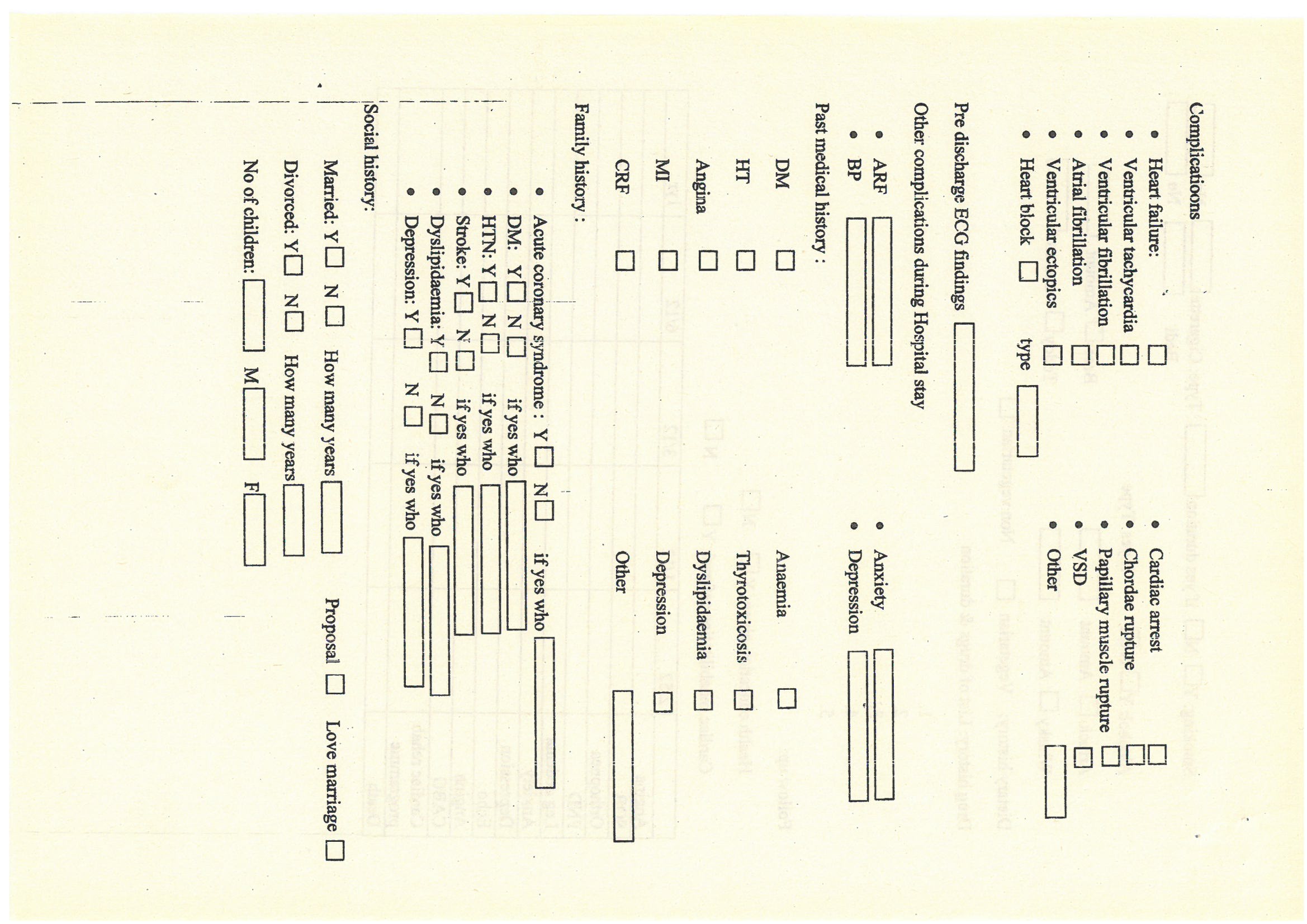

Supplement: Supplementary file 1 — Additional file 1: The English Version of the Questionnaire used for data collection. [file 12872_2019_1217_MOESM1_ESM.zip › Q Page 6R3.jpg]
